# Premiering pre-registration at *PLOS Biology*

**DOI:** 10.1371/journal.pbio.3001611

**Published:** 2022-03-31

**Authors:** Nonia Pariente, on behalf of the PLOS Biology Staff Editors

**Affiliations:** Public Library of Science, San Francisco, California, United States of America and Cambridge, United Kingdom

## Abstract

Pre-registration promises to address some of the problems with traditional peer-review. As we publish our first Registered Report, we take stock of two years of submissions and the future possibilities of this approach.

Two years ago, *PLOS Biology* launched Pre-Registered Research Articles (PRAs; more widely known as Registered Reports) as a new article type [1, 2]. Fast-forward through two years of research, a pandemic and a baby (welcomed by two of the main authors), and this month we have published our first PRA [3]. We thought it would be a good moment to take stock and reflect on what the fuss is about and what lessons we have learned along the way.

As editors, we know all too well that seemingly very interesting work can fall flat on its face because the experiments do not conclusively justify the claims and this problem arises when it is too late to intervene. Unlike regular research articles, PRAs undergo peer review immediately after the study design stage and before experiments are conducted. If these so-called “Stage 1” PRAs are issued an accept-in-principle decision, then the research is performed and results and discussion are added to create a single, fully integrated “Stage 2” article. The Stage 2 is assessed on its adherence to the approved approach, the appropriateness of any deviations, and the accuracy of the conclusions. This approach aims to minimise bias towards beautiful, unequivocal (and often cherry-picked and irreproducible) results, while also maximising study quality. It focuses peer review on the conclusiveness and rigour of the proposed methodology, rather than the perceived “coolness” of the results, when there is still time to do something about it. Our launch of PRAs coincided with a more general change in how we editorially assess all submissions to *PLOS Biology*, with a focus on the importance of the research question being asked, the approach used and the rigor of the execution, reducing the emphasis on the results obtained [1]. Launching PRAs at this time was therefore a natural fit.

To date, we have received 48 PRA submissions, of which 23% have been peer-reviewed, a figure that is in line with our journal average. However, of the articles that we have peer-reviewed, 88% received an accept-in-principle decision. This proportion is substantially higher than our average of approximately 50% and suggests that assessing the work at an early stage decreases the publication bias that arises from judging the perceived advance of the results. All of the Stage 1 PRAs that we have accepted underwent a major revision that led to modifications in the proposed study design and/or analysis. Thus, external expert input at this stage of the process clearly has value and should lead to more robust articles, as was suggested by a recent analysis of registered report research quality [4]. Over the past two years, we have engaged with many reviewers and Academic Editors who were unfamiliar with the PRA format, which has been an interesting experience. Some people are confused at first, finding reviewers is generally more difficult and they tend to take more time to review a manuscript as they familiarise themselves with the concept. However, most have become supportive and are even excited about the possibilities that this format affords. The majority of our PRAs are assigned two Academic Editors; one who oversees compliance with the PRA criteria and process and one who has relevant expertise on the topic of the work—the PRA that we have published this month is unusual in that, owing to the topic, one Academic Editor was able to fulfil both roles.

The PRA approach has the potential to be a game changer for the quality of published research, but which fields are seeing uptake? It is perhaps unsurprising that over 40% of our PRA submissions, including the first [3], are in neurobiology and behavioural science, as this is where the PRA format is better known. However, with the caveat that our numbers are small, we have received submissions in 10 different fields that are as wide-ranging as ecology, cell biology and meta-research, with virology and epidemiology being the most represented after neurobiology. This breadth was nice to see, as we believe that there is research from all walks of the life sciences that could be a good fit for a PRA. The format lends itself particularly well to hypothesis-driven research, where the question and the way to tackle it are apparent at the beginning of a project. Nevertheless, exploratory research could also be adapted to the PRA format [5]. Our first PRA combined the best of both worlds, as the authors first collected a discovery sample that was used to refine and develop the central hypotheses, which were then pre-registered and statistically tested with a confirmation sample.

Registered reports have the potential to make the publication process more effective by coupling grant and article review. Stage 1 manuscripts are similar to grant proposals in many ways, and turning a proposal into a Stage 1 manuscript is relatively straightforward. There have been several “one funder–one journal” trials, such as those between *PLOS ONE* and the Children’s Tumor Foundation, *PLOS ONE* and the Flu Lab or Cancer Research UK (CRUK) and the journal Nicotine & Tobacco Research [6]. Now, *PLOS Biology* and *PLOS ONE* are participating with 11 other journals in a Registered Reports Funding Partnership that is open to the applicants of two of CRUK’s Project Awards—Early Detection and Diagnosis Research and Prevention and Population Research. This consortium between one funder and multiple journals will give grantees a breadth of publication venues to choose from if they would like to submit a Stage 1 manuscript that arises from their funded grant application. Being familiar with the proposal, grant reviewers are likely to have constructive comments beyond those made during the grant assessment, and having an early commitment to publish the work regardless of the results would streamline the path from conceptualizing the research to its eventual publication.

Scientific publishing is in a state of transformation. Publishing PRAs and initiatives such as the CRUK Registered Reports Funding Partnership allow us to explore new ways of driving positive change. We hope that this PRA is the first of many and welcome their submission from all fields in the life sciences. If you are interested in this approach, we would love to hear from you!
